# Evaluation of Antileishmanial Activity of Selected Brazilian Plants and Identification of the Active Principles

**DOI:** 10.1155/2013/265025

**Published:** 2013-06-09

**Authors:** Valdir Cechinel Filho, Christiane Meyre-Silva, Rivaldo Niero, Luisa Nathália Bolda Mariano, Fabiana Gomes do Nascimento, Ingrid Vicente Farias, Vanessa Fátima Gazoni, Bruna dos Santos Silva, Alberto Giménez, David Gutierrez-Yapu, Efrain Salamanca, Angela Malheiros

**Affiliations:** ^1^Programa de Pós-Graduação em Ciências Farmacêuticas (PPGCF), Universidade do Vale do Itajaí, Rua Uruguai, 458, Centro, 88302-202 Itajaí, SC, Brazil; ^2^Núcleo de Investigações Químico-Farmacêuticas (NIQFAR), Universidade do Vale do Itajaí, Rua Uruguai, 458, Centro, 88302-202 Itajaí, SC, Brazil; ^3^Instituto de Investigaciones Fármaco Bioquímicas (IIFB), Universidad Mayor de San Andrés (UMSA), La Paz, Bolivia

## Abstract

This study evaluated extracts, fractions, and isolated compounds from some selected Brazilian medicinal plants against strains of promastigotes of *Leishmania amazonensis* and *L. brasiliensis in vitro*. The cell viability was determined, comparing the results with reference standards. The dichloromethane fractions of the roots, stems, and leaves of *Allamanda schottii* showed IC_50_ values between 14.0 and 2.0 **μ**g/mL. Plumericin was the main active compound, with IC_50_ of 0.3 and 0.04 **μ**g/mL against the two species of *Leishmania* analyzed. The hexane extract of *Eugenia umbelliflora* fruits showed IC_50_ of 14.3 and 5.7 **μ**g/mL against *L. amazonensis* and *L. brasiliensis*, respectively. The methanolic extracts of the seeds of *Garcinia achachairu* and guttiferone A presented IC_50_ values of 35.9 and 10.4 **μ**g/mL, against *L. amazonensis*, respectively. The ethanolic extracts of the stem barks of *Rapanea ferruginea* and the isolated compound, myrsinoic acid B, presented activity against *L. brasiliensis* with IC_50_ of 24.1 and 6.1 **μ**g/mL. Chloroform fraction of *Solanum sisymbriifolium* exhibited IC_50_ of 33.8 and 20.5 **μ**g/mL, and cilistol A was the main active principle, with IC_50_ of 6.6 and 3.1 **μ**g/mL against *L. amazonensis* and *L. brasiliensis*, respectively. It is concluded that the analyzed plants are promising as new and effective antiparasitic agents.

## 1. Introduction

*Leishmania *are protozoan parasites responsible for a spectrum of diseases known as leishmaniasis. There are two main forms of leishmaniasis: cutaneous, characterized by skin sores; and visceral, which affects the internal organs (e.g., the spleen, liver, and bone marrow). This disease is classified as a neglected disease and is one of major public health problem, causing significant morbidity and mortality in various countries. Leishmaniasis is considered by the World Health Organization as one of six major infectious diseases, with a high detection rate and ability to produce deformities [1–3].

Currently, the treatment of this disease is based on a limited number of chemotherapeutic agents, which represent high toxicity and cost. The search for new medicinal agents has become extremely important. The higher plants are a very rich source of new and selective substances, with therapeutic potential against these ailments. There are approximately 250.000 plant species worldwide, of which only a fraction has so far been studied. However, much research is needed to identify plants as sources of drugs or their phytoconstituents. The World Health Organization also advocates the use of traditional medicine for the treatment of these tropical diseases [4–6].

Our research group participated in an Iberoamerican program (RIBIOFAR/CYTED/CNPq) to search for Brazilian plants with therapeutic potential to treat several diseases, including the so-called neglected diseases. Some species have been previously analyzed with respect to their chemical composition and biological properties. Now we are interested in exploring the potential of the crude plant extracts, semi-purified fractions, and chemically defined molecules, in terms of their activity against leishmaniasis.  Table 1 gives a list of the plants analyzed, that were selected based on factors related to the discovery of new drugs from the biodiversity, the abundance of these plants, and previous pharmacological studies conducted at our laboratories. The selected plants, *Allamanda schottii*, *Rapanea ferruginea*, *Eugenia umbelliflora*, *Garcinia achachairu*, and *Solanum sisymbriifolium*, have exhibited various biological properties, including anti-proliferative antinociceptive, anti-inflammatory, antimicrobial, and gastroprotective effects [7–15].

## 2. Material and Methods

### 2.1. Plant Material

Different parts (leaves, stems, and roots) of *Allamanda schottii* were collected in the city of Blumenau, in December of 2006. The fruits of *Eugenia umbelliflora* Berg were collected in June 2008, in the town of Itapema. *Rapanea ferruginea, *synonymously *Myrsine coriacea* (Ruiz and Pavon) Mez, was collected in Blumenau in July 2007. *Garcinia achachairu *was collected in Camboriú, in March 2007. Aerial parts of* Solanum sisymbriifolium* Lam. were collected in Itapema, in January 2006. All the species were identified or authenticated by Professor Oscar Benigno Iza (Universidade do Vale do Itajaí), and vouchers were deposited at the Barbosa Rodrigues Herbarium (HBR, Itajaí), under numbers HBR 52525 (*A. schottii*), VC-Filho 50 (*E. umbelliflora*), HBR 52715 (*R. ferruginea*), HBR 52637 (*M. coriacea*), and VC-Filho 037 (*S. sisymbriifolium*).

### 2.2. Preparation of Extracts and Fractions

The dried vegetal material (100 g) of each part (roots, stems, and leaves) of *A. schottii *was macerated with 95% ethanol at room temperature, for seven days. Solvent removal was carried out under reduced pressure at temperatures below 45°C, until the desired concentrations were achieved, in order to obtain the ethanolic extracts. Part of each extract was dissolved separately in methanol : water (90 : 10) and successively partitioned with hexane, dichloromethane, and ethyl acetate to obtain the respective fractions, after the removal of solvents. The samples were stored under refrigeration and protected from light until analysis.

The fruits of *E. umbelliflora* were dried under air circulation (37°C) for 2 days and powdered using a knife grinder. The dried fruits (570 g) were extracted twice by maceration with *n*-hexane for five days. Next, solvent was removed under vacuum to yield a dry residue of 2.62% hexane extract. The fruits were then reextracted using dichloromethane and methanol, following the methods described above. The separate extracts were submitted to concentration to obtain dichloromethane and methanolic extract 1 with yields of 1.16% and 4.47%, respectively. The methanolic extract 2 was obtained by extracting the dried fruits with methanol at room temperature for seven days.

The dried material 100 g of each part (leaves, stem barks and fruits) of *R. ferruginea *was macerated with 95% ethanol at room temperature, for seven days. Solvent removal was carried out under reduced pressure at temperatures below 45°C, until the desired concentrations were achieved, in order to obtain ethanolic extracts with yields of 16%, 15%, and 13% from leaves, stem, and fruits, respectively. The chloroform extract was obtained by the stem barks and is described in Hess et al. [9].

The air-dried and powdered seeds, leaves and branches (250 g each) of *G. achachairu* were separately extracted at room temperature with methanol for seven days. The macerated material was filtered and concentrated under reduced pressure, yielding 9.01 g (3.6%), 15.0 g (6%), and 12.0 g (4.8%) of crude methanolic extract, respectively. 

The powdered leaves, and stem (680.0 g) of *S. sisymbriifolium* were extracted with MeOH for ten days. The concentrated methanolic extract was diluted with a water : methanol mixture (9 : 1, 300 mL) and extracted with hexane (11.5 g; 1.70%), chloroform (11.2 g; 1.64%), and ethyl acetate (1.18 g; 0.17%). 

### 2.3. Isolation and Identification of Components

*A. schottii*. The ethanolic extract of *A. schottii* stems (15.1 g) was chromatographed in a silica-gel column with a hexane: ethylacetate: ethanol gradient. The fraction eluted with *n*-hexane : ethyl acetate 7 : 3 yielded plumericin (15.2 mg). Elution with a hexane : ethyl acetate ratio of 6.5 : 3.5 yielded ursolic acid (62.4 mg). The fraction eluted with ethyl acetate : ethanol 8 : 2 yielded plumieride (39.4 mg) as described previously by Schmidt et al. [7] and Malheiros et al. [29].

*R. ferruginea*. The CHCl_3_ extract of *R. ferruginea* (63.4 g) was subjected to column chromatography packed with silica gel 60–230 mesh and eluted with hexane gradually enriched in ethyl acetate. The fraction eluted with 40% ethyl acetate in hexane provides impure myrsinoic acid B. This fraction (8.5 g) was chromatographed in a silica-gel column with hexane : ethyl acetate to provide pure myrsinoic acid B (4.3 g) as described previously by Baccarin et al. [30]. 

*E. umbelliflora*. The complete procedure used to obtain the compound isolated from *E. umbelliflora* fruits, the meroterpenoid Eugenial A, was described previously by Faqueti et al. [11]. 

*G. achachairu*. The methanolic extract of the seeds (5.0 g) was chromatographed on a silica-gel column (0.063–0.20 mm, 84.0 g, 2.5 × 50 cm, Merck) and eluted with a gradient of CHCl_3_–MeOH (100 -> 0) yielding guttiferone A as described previously by Dal Molin et al. [13].

**Figure 1:**
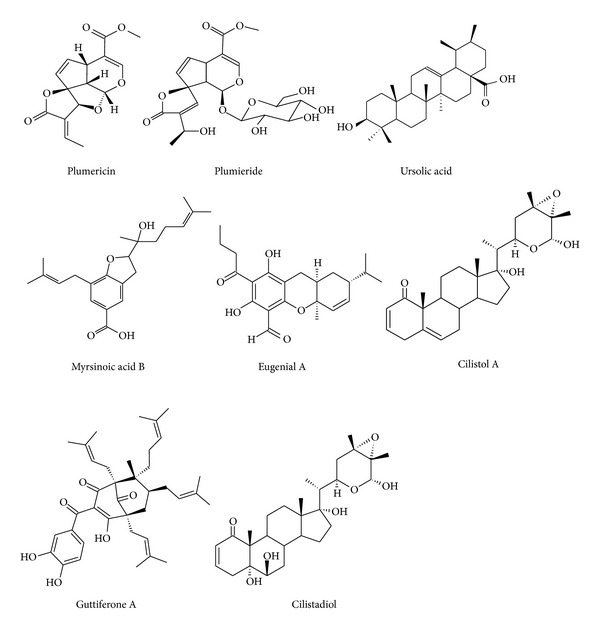
Molecular structure of the isolated compounds from analyzed plants.

*S. sisymbriifolium*. The chloroform fraction was purified by CC on silica gel, yielding Cilistol A (60.0 mg) and cilistadiol (25.0 mg) as described previously by Niero et al. [8]. The molecular structures of isolated compounds are presented in Figure 1.

### 2.4. Leishmanicidal Activity

#### 2.4.1. Promastigotes of *Leishmania amazonensis* Clone 1

AML (MHOM/BR/76/LTB-012) and *Leishmania braziliensis* (M2904 C192 RJA) obtained from *in vitro* cultures of IIFB (20 *μ*L) were fixed with glutaraldehyde (5%, 180 *μ*L) and counted in a Neubauer chamber. The population was adapted to 3 × 10^6^ parasites/mL with Schneider medium (pH = 6.8) and Fetal Bovine Serum (10%), distributed (100 *μ*L/well) in 96-microwell plates. Solutions of the samples, at different concentrations, were added (100 *μ*L). DMSO (1%) and Amphotericin B (0.5 *μ*g/mL) were used as parasite growth control. Each test was performed in triplicate and the plates were incubated for 72 hours at 26°C.

To each well of the plate was added (50 *μ*L/well) a solution of XTT (1 mg/mL) in phosphate buffer (pH 7.0, 37°C) with PMS (Sigma-Aldrich, 0.06 mg/mL), which was incubated for 4 h at 26°C. The plates were read on a computer Stat Fax (Model 2100 Series-Plate Reader) at 450 nm. The IC_50_ values of 50% inhibitory concentration of parasites were calculated using Microsoft Excel 2000 [31].

### 2.5. Statistical Analysis of Data

The samples were analyzed in replicates with *n* = 4, using two methods of viability assessment (XTT and Neutral Red). The data are mean ± stand error, and statistical differences were determined by the one-way ANOVA followed by Tukey-Kiover (*P < 0.05, **P < 0.01 and ***P < 0.001).

## 3. Results and Discussion

In this work we tested extracts, fractions, and compounds of five Brazilian medicinal plants to determine their *in vitro *antiparasitic effect against promastigotes of *L. amazonensis* and *L. brasiliensis*. For the initial screening, extracts with IC_50_ less than 100 *μ*g/mL were considered active. The results of the minimal inhibitory concentrations are shown in Tables 2 and 3.

The ethanolic extracts from different parts of *A. schottii* (roots, stems, and leaves) exhibited pronounced leishmanicidal activity (Table 2). The best results were obtained from the root extracts with IC_50_ of 43.8 *μ*g/mL for *L. amazonensis* and 8.5 *μ*g/mL for *L. brasiliensis*. The extracts were submitted to partition with hexane, dichloromethane, and ethyl acetate. The activity was concentrated in the dichloromethane fraction for all extracts with IC_50_ of 2.1 to 13.4 *μ*g/mL. The best activity was observed in the dichloromethane fraction of the roots with IC_50_ of 2.1 *μ*g/mL for *L. amazonensis* and 8.8 *μ*g/mL for *L. brasiliensis*, suggesting that the iridoid plumericin and the triterpene ursolic acid are the main active principles. These compounds were isolated from the ethanolic extract of the stems and detected in all the dichloromethane fractions by thin layer chromatography. Plumericin presented IC_50_ of 0.3 *μ*g/mL (0.98 *μ*M) for *L. amazonensis* and 0.04 *μ*g/mL (0.13 *μ*M) for *L. brasiliensis*. Ursolic acid was active against the two species evaluated with IC_50_ of 66.1 *μ*g/mL and 8.3 *μ*g/mL for *L. amazonensis* and *L. brasiliensis*, respectively. Amphotericin B, used as positive control, exhibited activity with IC_50_ of 0.6 *μ*g/mL and 0.7 *μ*g/mL, respectively, for the two species evaluated (Table 3). Another evaluated iridoid was plumieride, a glycoside iridoid, which was detected in all the ethyl acetate fractions, presenting IC_50_ of 21.3 *μ*g/mL for *L. brasiliensis*. This compound may be responsible for the activity observed in ethyl acetate fractions of different parts of this plant. 

The Apocynaceae family is as a rich source of species with antileishmanicidal activity and for some species, plumericin is the main active compound. This compound, previously isolated from *Himatanthus sucuuba*, presented IC_50_ values of 0.9 and 1.0 *μ*M against promastigote and amastigote forms of *L. amazonensis* [32] and IC_50_ of 3.17 ± 0.12 and 1.41 ± 0.03 *μ*M against promastigotes and amastigotes of *L. donovani*, respectively [33].

On the other hand, ursolic acid was isolated as an active compound from extracts of *Baccharis dracunculifolia* (Asteraceae) against *Leishmania donovani *with IC_50_ of 3.7 *μ*g/mL [34]. Our results, together with those reported in the literature, suggest that the compounds found in *Allamanda genus *are promising antileishmanial agents.

The antiparasitic investigation against promastigotes of *L. amazonensis* and *L. brasiliensis *with respect to *E. umbelliflora* fruits indicated that the hexane extract was the most active with IC_50_ of 14.3 ± 0.86 and 5.7 ± 0.92 *μ*g/mL, respectively. When the fruits were directly submitted to methanol extraction, activity was observed with IC_50_ of 12.5 ± 0.5 and 7.8 ± 0.4 *μ*g/mL against *L. amazonensis *and *L. brasiliensis*, respectively. Despite reports in the ethnobotanical literature on the medicinal uses of *Eugenia* species to treat some diseases, there have been few scientific studies that validate their antileishmanial activity. Previous studies with *Eugenia *genus have suggested that terpenic compounds found in the essential oil may have potential anti-leishmanial activity [35].

Continuing our screening program in the search for bioactive molecules from Brazilian plants, we have investigated *G. achachairu *for its leishmanicidal activity. Thus, the methanolic extract, some fractions, and isolated compounds were evaluated. As can be seen in Table 2, the methanolic extract of the seeds, leaves, and branches obtained from* G. achachairu* exhibited IC_50_ values of 35.9 and 100 *μ*g/mL in promastigote forms of *L. amazonensis* and 28.2, 100, and 81.6 *μ*g/mL against promastigote of *L. brasiliensis*, respectively. The most bioactive extract (from the seeds) was chromatographed on a silica-gel column yielding guttiferone A as the main constituent. This compound, after evaluation in promastigote forms of *L. amazonensis* and *L. brasiliensis* (Table 3), showed a significant activity with IC_50_ values of  10.4 and 18.4 *μ*g/mL. 

The ethanolic extracts of fruits, leaves, and stem bark of *R. ferruginea* were evaluated against promastigotes of *L. amazonensis* and *L. brasiliensis*. Only the extracts of the stem bark presented activity, with IC_50_ of 66.4 *μ*g/mL for *L. amazonensis* and 24.9 *μ*g/mL for *L. brasiliensis*. In this extract, the main compound was a prenylated benzoic acid derivative known as myrsinoic acid B, with IC_50_ of 24.1 *μ*g/mL for *L. amazonensis* and 6.1 *μ*g/mL for *L. brasiliensis*. These extracts were previously evaluated by high performance liquid chromatography, and myrsinoic acid B was confirmed as the main compound in the stem bark extract. In leaves and fruits, it is present in lower concentrations [30]. It is interesting to note that species from the *Rapanea* genus are not known for their leishmanicidal activity, our studies being the first to suggest its importance as a possible source of antileishmanicidal agents.

According to the literature data, some biological activities of natural products, such as antimicrobial and trypanocidal products, are associated with their prenylated compounds, which may be increased by increasing the number of prenyl residues attached by an increase in lipophilicity [36, 37]. Thus, considering that guttiferone A and myrsinoic acid B are prenylated compounds, the activity observed for these compounds could be explained.

Regarding *S. sisymbriifolium*, we have observed an important antileishmanial activity. As shown in Table 2, the chloroform fraction exhibited IC_50_ values of 33.8 *μ*g/mL against promastigote forms of *L. amazonensis*. On the other hand, the hexane and ethyl acetate fractions exhibited IC_50_ values of 100 *μ*g/mL. In relation to the promastigote forms of *L. brasiliensis*, both hexane and chloroform fractions presented IC_50_ values of 74.3 and 20.50 *μ*g/mL, respectively. However, the ethyl acetate fractions exhibited an IC_50_ value of 100 *μ*g/mL, suggesting that the less polar compounds are responsible for the observed activity. This can be confirmed by the activity of two compounds isolated from the chloroform fractions and identified as cilistol A and cilistadiol (Figure 1). These compounds exhibited IC_50_ values of 6.60 and more than 100 *μ*g/mL against the promastigote forms of *L. amazonensis *and IC_50_ values of 3.1 and 59.8 *μ*g/mL for the promastigote forms of *L. brasiliensis*, respectively (Table 3).

## 4. Conclusions

Our results, together with those reported in the literature, strongly suggest that *Allamanda schottii*, *Rapanea ferruginea*, *Eugenia umbelliflora*, *Garcinia achachairu*, and *Solanum sisymbriifolium* and, in particular, the compounds plumericin, plumieride, ursolic acid, guttiferone A, cilistol A, and cilistadiol could be promising for the treatment of leishmaniasis caused by protozoans, demanding a search for new chemotherapeutic agents. However, further studies (*in vitro* and *in vivo*) need to be carried out, in order to understand the mechanisms of action and to evaluate the toxicity, searching for a clinical use for these bioactive compounds.

## Figures and Tables

**Table 1:** Traditional uses of the selected Brazilian medicinal plants and previous pharmacological studies.

Species	Popular use	Biological studies	Reference
*A. schotii *	Latex: used as scabicide and in louse control The infusion of flowers is purgative and antihelminthic The stems are used against hepatic tumors	antiproliferative effect leukemic cells	[7, 16–20]

*E. umbelliflora *	Fruits and leaves used to treat various ailments such as infections, inflammation, and diabetes	Antimicrobial, gastroprotective, antifungal	[10, 21–23]

*G. achachairu *	Rheumatism, inflammation, pain, and gastric disorders	Low genotoxicity, gastroprotective, and antinociceptive activity	[13–15, 24]

*R. ferruginea *	The leaves or the bark prepared as a tea is indicated as a diuretic, to combat diseases of the urinary tract and also is a good cleanser. It is used too for itching, rashes, hives, eczema, rheumatism, and diseases of the liver	Anti-inflammatory and analgesic activity	[9, 12, 25]

*S. sisymbriifolium *	Diarrhea, respiratory and urinary infections	Hypotensive and molluscicidal activity	[26–28]

**Table 2:** *In vitro* leishmanicidal activity of extracts on promastigotes of *Leishmania amazonensis *and *L. brasiliensis*.

Species	Sample	*L. amazonensis *IC_50_ (*μ*g/mL)^a^	*L. brasiliensis *IC_50_ (*μ*g/mL)^a^
*A. schottii *	Ethanolic extract roots	43.8 ± 1.13	8.5 ± 0.47
	Hexane fraction	49.4 ± 2.27	48.6 ± 1.23
	Dichloromethane fraction	2.1 ± 0.25	8.8 ± 0.37
	Ethyl acetate fraction	85.6 ± 3.57	67.8 ± 0.14
	Ethanolic extract stems	>100	39.0 ± 0.56
	Hexane fraction	>100	17.8 ± 1.83
	Dichloromethane fraction	13.6 ± 3.80	8.2 ± 0.07
	Ethyl acetate fraction	>100	47.9 ± 3.60
	Ethanolic extract leaves	63.8 ± 1.10	65.7 ± 6.71
	Hexane fraction	>100	36.7 ± 6.80
	Dichloromethane fraction	13.4 ± 1.27	8.9 ± 0.63
	Ethyl acetate fraction	>100	32.9 ± 6.10

*E. umbelliflora *	Hexane extract fruits	14.3 ± 0.86	5.7 ± 0.92
	Dichloromethane extract fruits	37.0 ± 1.02	20.7 ± 0.05
	Ethyl acetate extract fruits	27.2 ± 4.80	37.6 ± 0.44
	Methanolic extract fruits 1	>100	>100
	Methanolic extract fruits 2	12.5 ± 0.50	7.8 ± 0.40

*G. achachairu *	Methanolic extract seeds	35.9 ± 0.52	28.2 ± 0.35
	Methanolic extract leaves	>100	>100
	Methanolic extract branches	>100	81.6 ± 0.25

*R. feruginea *	Ethanolic extract fruits	>100	>100
	Ethanolic extract leaves	>100	>100
	Ethanolic extract stem barks	66.4 ± 2.68	24.9 ± 1.79

*S. sisymbriifolium *	Hexane fraction	>100	74.3 ± 0.22
	Chloroform fraction	33.8 ± 0.81	20.5 ± 0.76
	Ethyl acetate fraction	>100	>100

Positive control	Catetanol	21.1 ± 4.27	21.4 ± 5.53
	Amphotericin B	0.6 ± 0.36	0.7 ± 0.36

^a^Data are expressed as mean ± standard deviation of three determinations.

**Table 3:** *In vitro* leishmanicidal activity of isolated compounds on promastigotes of *Leishmania amazonensis *and *L. brasiliensis*.

Species	Isolated compounds	*L. amazonensis* IC_50_ (*μ*g/mL)^a^	*L. brasiliensis* IC_50_ (*μ*g/mL)^a^
*A. schottii *	Plumericin	0.3 ± 0.07	0.04 ± 0.007
	Plumieride	>100	21.3 ± 2.80
	Ursolic acid	66.1 ± 1.22	8.3 ± 0.84

*R. ferruginea *	Myrsinoic acid B	24.1 ± 0.52	6.1 ± 0.24

*E. umbelliflora *	Eugenial A	>100	53.8 ± 1.71

*G. achachairu *	Guttiferone A	10.4 ± 0.50	18.4 ± 0.20

*S. sisymbriifolium *	Cilistol A	6.6 ± 0.22	3.1 ± 0.25
	Cilistadiol	>100	59.8 ± 0.32

Positive control	Catetanol	21.1 ± 4.27	21.4 ± 5.53
	Amphotericin B	0.6 ± 0.36	0.7 ± 0.36

^a^Data are expressed as mean ± standard deviation of three determinations.
